# Quality investigation of hydroxyprogesterone caproate active pharmaceutical ingredient and injection

**DOI:** 10.3109/03639045.2012.662511

**Published:** 2012-02-13

**Authors:** John L. Chollet, Michael J. Jozwiakowski

**Affiliations:** Ther-Rx Corporation, St. Louis, MO, USA

**Keywords:** Hydroxyprogesterone caproate, injection, compounding pharmacy, preterm birth

## Abstract

The purpose of this study was to investigate the quality of hydroxyprogesterone caproate (HPC) active pharmaceutical ingredient (API) sources that may be used by compounding pharmacies, compared to the FDA-approved source of the API; and to investigate the quality of HPC injection samples obtained from compounding pharmacies in the US, compared to the FDA-approved product (Makena®). Samples of API were obtained from every source confirmed to be an original manufacturer of the drug for human use, which were all companies in China that were not registered with FDA. Eight of the ten API samples (80%) did not meet the impurity specifications required by FDA for the API used in the approved product. One API sample was found to not be HPC at all; additional laboratory testing showed that it was glucose. Thirty samples of HPC injection obtained from com pounding pharmacies throughout the US were also tested, and eight of these samples (27%) failed to meet the potency requirement listed in the USP monograph for HPC injection and/or the HPLC assay. Sixteen of the thirty injection samples (53%) exceeded the impurity limit setforthe FDA-approved drug product. These results confirm the inconsistency of compounded HPC Injections and suggest that the risk-benefit ratio of using an unapproved compounded preparation, when an FDA-approved drug product is available, is not favorable.

## Introduction

**Figure 1:**
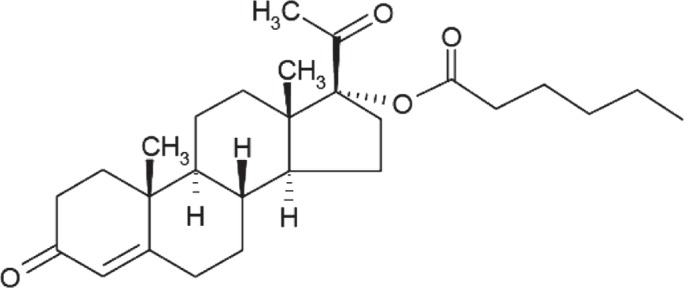
Chemical structure of hydroxyprogesterone caproate.

Babies born prematurely have higher rates of mortality and morbidity, and may have developmental difficulties later in life^1^. Risk factors for a woman having a preterm birth prior to 37 weeks of gestation include multiple gestations, short cervical length, and African-American descent. However, the subset of women who have experienced a prior spontaneous preterm birth are at an especially high risk of having another preterm birth^2^. Various forms of progesterone have been studied to reduce the risk of premature birth, using several different dosage forms and routes of delivery^3^. The Maternal-Fetal Medicine Units Network of the National Institute of Child Health and Human Development showed that treatment with a sterile injectable form of hydroxyprogesterone caproate (HPC, Figure 1) significantly reduced the risk of delivery at less than 37 weeks in this population: 36.3% in the HPC treatment group versus 54.9% in the placebo group^4^. The American College of Obstetricians and Gynecologists (ACOG) and the Society for Maternal-Fetal Medicine (SMFM) recommend the use of progesterone to reduce preterm birth to women with a singleton pregnancy and a history of spontaneous preterm birth^5^.

HPC was originally used for the treatment of threatened abortion and amenorrhea^6^, and was later used at higher doses for advanced endometrial cancer^7^. At the request of the sponsor, the drug was withdrawn from the US market in 2000 for commercial reasons. An FDA-approved source of HPC injection indicated to reduce the risk of preterm birth did not become commercially available until 2011, and so for several years physicians have used compounding pharmacies to source this medicine for their patients. Traditional pharmacy compounding is an important channel for provision of a customized medication that is not commercially available in a form suitable for an individual patient. This can include making suspensions of solid dosage forms for patients unable to swallow pills, or omitting an inactive ingredient to which the patient has an allergy. Parenteral products pose a major risk of microbial contamination if the compounding processes are not conducted under sterile conditions, but not all compounding pharmacies have appropriate facilities and procedures to make sterile injectable formulations. USP chapter <797> *Pharmaceutical Compounding - Sterile Preparations,* defines the practice of incorporating non-sterile ingredients into a terminally sterilized preparation as a high risk level procedure^8^. Nevertheless, prior to the availability of an FDA-approved source of HPC injection, the risk-benefit analysis favored using compounded preparations of HPC (often referred to as “17P”), since the opportunity to reduce the risk of a preterm birth outweighed the risk of possible formulation errors.

FDA-approved branded and generic drugs are subject to FDA regulations, and are produced at facilities that FDA periodically inspects for compliance with good manufacturing practices (GMP). In contrast, oversight of compounding pharmacies is a state function that is generally delegated to State Board of Pharmacy. Because of the inherent variability of drugs made by different entities to different standards, FDA and pharmacy professionals have warned against the risks of using compounded drugs that are produced without GMP controls, and the practice of compounding drugs for which an approved commercial alternative is available^9^,^10^. This is especially true for drugs used in high risk patient populations, such as pregnant women. ACOG has published a Committee Opinion^11^ stating that since significant quality concerns may exist for compounded agents, particularly when sterility is important such as with injectable drugs physicians and patients should exercise caution in prescribing and using products that are largely untested for safety and efficacy.

FDA considers compounded drugs to be “unap-proved new drugs” because their safety and efficacy has not been established through clinical trials. FDA has stated that poor quality compounded drugs are a serious public health concern. In one survey^12^, FDA sourced compounded drugs from across the country and analyzed them for potency. Of the 36 samples that were still within their expiry dating and had acceptable analytical data, 33% failed the testing, primarily due to potency issues (range 67.5-268.4% of label claim). The Missouri Board of Pharmacy has conducted similar testing, with “unsatisfactory” test results ranging from 11.6% to 25.2% of samples between 2006 and 2009^13^, and potency ranging from 0.0% to 450.4% of the label claim. The literature indicates that instances of compounded drugs failing to meet basic quality requirements are relatively common, with issues being found on products as diverse as simvastatin capsules^14^, amifampridine tablets and capsules^15^, nitroglycerin ointments^16^ and progesterone vaginal suppositories^17^. There are also numerous reports of serious adverse drug experiences caused by improperly prepared compounded drugs, either from contamination or potency errors^18^,^22^.

In February 2011, FDA approved the first commercial form of HPC injection (Makena®) for use in women with a singleton pregnancy and a previous history of spontaneous preterm birth, and granted it 7 years of orphan drug exclusivity. This approval was based in part on the results of the Meis study^4^, which used drug supplies made at GMP facilities, not drug from compounding pharmacies, in accordance with the FDA Regulations for Investigational New Drugs. Despite the fact that an FDA approved drug is available, some healthcare professionals have advocated the continued use of compounded 17P, because of the cost differential between FDA-approved GMP product (Makena®) and compounded 17P^23^. In response to the ensuing controversy, FDA issued a public statement on March 30, 2011 stating that the agency did not intend to take enforcement action against pharmacies that compound HPC based on a valid prescription for an individually identified patient^24^. This action by the FDA was contrary to its normal historical implementation of policies concerning orphan drug exclusivity and unapproved drugs^25^. In its statement, FDA characterized this as a unique situation, and stated that it may revisit the decision at anytime if it finds the compounded products to be unsafe, of substandard quality, or not being compounded in accordance with appropriate standards.

The purpose of the current investigation was to ascertain whether previous studies showing varying quality with compounded drugs also applied to compounded HPC injection, and to compare the quality of drug product samples obtained from compounding pharmacies to the approved product in terms of potency and impurity levels. Since the quality of the injection also depends on the quality of its ingredients, the potency and purity of the samples of active pharmaceutical ingredient (API) available for compounding HPC injections were also examined.

## Methods

### Sample procurement

Fuld and Company (Cambridge, MA), a research and consulting firm in the field of competitive intelligence, was contracted to investigate sources of HPC API available for use in the pharmacy compounding of HPC injections. Suppliers advertising the sale of HPC API for human use were identified through interviews of compounding pharmacists and their suppliers, or by searching chemical supplier websites (Alibaba, The Chemical Register, etc.) Test samples were requested from every available supplier confirmed to be an original manufacturer of HPC, as opposed to an importer, repackager, or broker. The registration status of each firm was verified from information provided online by FDA. All packaging and labeling was documented upon receipt of the sample, prior to shipment to the analytical testing laboratory. Vials of HPC injection were ordered for office use from compounding pharmacies by a licensed healthcare professional, and then transferred to The Coghlan Group (Bastrop, TX), a licensed wholesale pharmaceutical distributor. Thirty compounding pharmacies of varying sizes in 15 different US states provided samples of HPC injection. Every sample that was received was tested without any further selection criterion. Samples were stored at The Coghlan Group's facility at controlled room temperature in locked cabinets prior to transfer to the contract analytical laboratories for testing.

Testing for this investigation was performed by two independent contract test laboratories that were selected by Fuld and Company (Fuld) and The Coghlan Group (TCG) for their expertise and capabilities in pharmaceutical analysis: Cerilliant Corporation (Round Rock, TX) and Chemir Analytical Services (Maryland Heights, MO). Fuld and TCG shipped the samples directly from their sites to the testing laboratories under controlled chain of custody, supervised the testing, and received the final results directly from the testing facilities. The study sponsor, Ther-Rx Corporation, commissioned the testing, but did not perform any analyses, and did not at any point have custody of any sample that was tested in this study, except for control samples that it provided of the approved API and approved drug product (Makena®).

### Test methods

The current USP monograph for HPC API includes specifications and standardized USP test procedures for identification *(InfraredAbsorption, USP <197K>),* meltingrange *(USP <741>),* specific rotation *(USP <781 S>),* water *(USP <921> Method I),* free caproic acid (by titration), ordinary impurities *(USP <466>),* and assay (by UV absorbance). Residual solvents *(USP <467>)* is a general test in the USP that is required for all drug substances and drug products, although it is not listed in individual monographs. In addition to the USP monograph tests, the FDA-approved specifications for the API that is used in the approved HPC injection drug product (Makena®) include the following tests: appearance (visual examination), identification (TLC), assay procedure (HPLC), related substances (HPLC), and loss on drying. The complete specifications for the approved API and injection product are summarized in Tables 1 and 2.

**Table 1:** FDA approved specifications for hydroxyprogesterone caproate API.

Test	Acceptance criteria	Test origin	Tested in this investigation
Identification infrared absorption <197K>	Matches reference spectrum	USP	Yes
Melting range <741>	120-124°C	USP	Yes
Specific rotation <781S>	+58 to +64°	USP	No
Water, <921> Method 1	NMT 0.1%	USP	No
Free n-caproic acid (titration)	NMT 0.58%	USP	No
Ordinary impurities (TLC)	NMT 2.0%	USP	Yes
Assay (UV)	97.0-103.0%	USP	Yes
Residual sovents	Meets USP <467> requirements	USP	Yes
Appearance	White to practically white powder or crystals with no visible impurities	Manufacturer	Yes
ID (TLC)	Matches reference standard	Manufacturer	No
Loss on drying	≤0.5%	Manufacturer	No
Assay (HPLC)	97.0-103.0%	Manufacturer	Yes
Related substances (HPLC)		Manufacturer	Yes
17a-hydroxyprogesterone	NMT 1.0%		
17a-|3-methyl-d-homo compound	NMT 0.2%		
Any unspecified impurity	NMT 0.10%*		
Total impurities	NMT 2.0%		

* API manufacturer's specification limit for unspecified impurities, which is equivalent to FDA Q3A identification threshold for impurities in new drug substances, when maximum daily dose ≤2g.

**Table 2:** Hydroxyprogesterone caproate injection - specifications.

Test	Acceptance criteria	Tested in this investigation
USP monograph		
Identification Test A (wet chemical test)	Conforms (color change)	No
Identification Test B (TLC)	Conforms (sample matches standard)	No
Water: USP <921>	NMT 0.2%	No
Assay (UV)	90.0%-110.0%	Yes
FDA approved specifications for Makena		
Appearance	Clear, yellow color, essentially free of foreign particulate matter, viscous and oily solution with an organic odor	Yes
ID (HPLC peak retention time)	Retention time of sample matches standard	Yes
Assay (HPLC)	90-11%	Yes
Purity (HPLC)	NLT 98.0%	Yes
17α-hydroxyprogesterone	NMT 1.0%	
Any unspecified impurity	NMT 0.2%*	
Water: USP <921>, Method 1	NMT 0.2%	No
Benzyl alcohol (HPLC)	1.7-2.3%	No
Volume in container: USP <1>	NLT 5.0 mL/vial	No
Particulate Matter: USP <788>, Test 1B light obscuration particle count test	≥10 micron: NMT 6000 particles/vial; ≥25micron: NMT 600 particles/vial	No
Sterility: USP <71>	Product is sterile	No
Bacterial Endotoxins: USP <85>	NMT 0.5 EU/mg	No

* Approved drug product manufacturer's specification limit for unspecified impurities, which is equivalent to FDA Q3B identification threshold for impurities in new drug products, when maximum daily dose is <10 mg-2 g.

In this investigation, selected tests were performed to assess the quality of different sources of HPC. The testing included the USP procedures for identification (IR), melting range, ordinary impurities (TLC), assay (UV), and residual solvents, and the non-USP procedures for appearance, assay (HPLC), and related substances (HPLC). Although elemental impurities is not currently included in the API specifications or the current USP monograph, the API samples were also tested for toxic metals by the proposed USP <233> procedure using inductively coupled plasma/mass spectrometry (ICP/ MS). Several tests in the specifications for the approved API were not performed, because it was considered unlikely that these tests (e.g. loss on drying) would provide information useful in terms of discriminating between the quality of the various API samples. Due to sample size limitations, testing of the drug product in this investigation primarily focused on evaluation of identity, content assay, and purity, plus a visual assessment of the product's appearance. The small sample size precluded microbiological testing of the injection samples for sterility and bacterial endotoxins, and physical testing for particulate matter. API content was assayed by both the non-specific USP method (UV), which compounding pharmacies may use to test HPC injections, and the manufacturer's proprietary validated HPLC procedure, which is more specific than the USP method. Similarly, the USP test for “ordinary impurities” (TLC) is less specific and less sensitive than the manufacturer's validated HPLC procedure for related substances, so both the TLC and the HPLC procedures were performed.

At the present time, the USP is actively engaged in efforts to modernize official USP-NF monographs. The goal is to add new procedures to assess critical quality attributes such as purity to monographs that lack them, and to replace test methods in monographs that utilize outdated technology (such as UV and TLC) with modern procedures (such as HPLC). The current list of USP monographs in need of modernization includes both the HPC monograph and the HPC injection monograph.

### Sample analysis

#### HPLC Assay and Purity

Isocratic reversed-phase HPLC methods were used to test content and purity of the HPC API and the HPC injection. The test procedures were validated, and had previously been accepted by the FDA in the registration dossiers for the approved HPC API and HPC injection (Makena®). The independent laboratories that performed the testing qualified the procedures by testing control samples of the approved HPC API and HPC injection, and comparing their results to the certificates of analysis prior to testing unknown samples. Both testing laboratories used Agilent 1100 HPLCs, with variable wavelength detectors. Solutions were maintained in a chilled autosampler at 6°C throughout the analyses.

For the API analysis, a Nova-PakC12 Radial-Pak(4 μm), length 10 cm, internal diameter 8 mm column was used with a Waters radial compression module, WAT 082887. Sample and standard solutions were prepared at a concentration of 300 μg/mL in methanol. Mobile phase was composed of methanol and water in a ratio of 82:18 (v/v), at a flow rate of 1.7 mL/min. Detection wavelength was 254 nm, with a run time of 20 min. Injection volume was 20 μL.

For HPC injection analysis, a Waters Symmetry C18, (5 μm), length 25 cm, internal diameter 4.6 mm column was used. Standard solutions were prepared at a concentration of 50 μg/mL hydroxyprogesterone caproate in methanol. Potency assay sample solutions were prepared at a concentration of 50 μg/mL hydroxyprogesterone caproate in methanol; purity sample solutions were prepared at a concentration of 250 μg/mL of hydroxyprogesterone caproate in methanol. Mobile phase was composed of methanol and water in a ratio of 80:20 (v/v), at a flow rate of 1.0 mL/min. Detection wavelength was 242 nm, with a run time of 20 min. Injection volume was 20 μL.

#### Other Analyses

UV assays were performed per the USP Assay procedure for Hydroxyprogesterone Caproate and the USP Assay procedure for Hydroxyprogesterone Caproate Injection using a Perkin Elmer Lambda 35 UV-Vis spectropho-tometer and an Agilent 8453 UV-Vis spectrophotometer. Melting points were determined per USP General Test <741> using a Mettler Toledo model FP62 melting point apparatus. Identification (ID) testing was performed per USP General Test <197K> using a Thermo Scientific Nicolet 6700 Fourier Transform Infrared Spectrometer (FTIR). Residual solvent testing was performed per USP General Test <467> using an Agilent G1530A gas chro-matograph with Flame Ionization Detection (GC-FID).

Additional structural identification of the API sample that was not HPC was achieved using a Surveyor HPLC with Thermo/Finnigan LCQ Advantage Mass Spectrometer (LC/MS) and a Varian Mercury Plus, 400 MHz nuclear magnetic resonance spectrometer (NMR).

## Results

### HPC API

A total of 11 HPC API samples were analyzed, including one lot from the FDA-registered supplier in Europe, seven from original manufacturers of bulk product in China, and three from repackagers located in the United States. A control sample (manufacturer's lot # L0031857) of the source of API used in the approved drug product, which came from an FDA-registered supplier in Europe, was provided by the sponsor. More than 50 potential sources of HPC API available for the compounding of HPC injection were initially located. Most of these sources were brokers who import and repackage drugs, rather than original manufacturers who synthesize the API. A total of seven suppliers that synthesize the API and were willing to export test samples to the United States (Table 3, Manufacturers A-G) were located. These suppliers were all facilities in China that did not have the required FDA registration and have not been FDA inspected. Additionally, three API samples that had been repackaged and relabeled by US resellers, were also tested (Table 3, Reseller A-C). The test samples that were provided came shipped in glass bottles, plastic jars or sealed plastic bags. Only three of the ten suppliers provided certificates of analysis with the API.

**Table 3:** Hydroxyprogesterone caproate API - laboratory test results.

Sample source	Identity (IR, TLC)	Melting point (°C)	UV assay (%)	HPLC assay (%)	Unspecified impurities < 0.10%	% Total impurities (HPLC)
Control	Pass	122	99.7	99.8	none	0.19
MFR. A	Pass	121	99.4	99.2	RRT 0.36: 0.12%* RRT 0.48: 0.24%*	0.67
MFR. B	Pass	121	99.8	98.9	RRT 0.36: 0.11%* RRT 0.48: 0.26%*	0.67
MFR. C	Pass	121	101.3	99.7	None	0.47
MFR. D	Pass	121	101	99.8	RRT 0.36: 0.14%* RRT 0.48: 0.12%*	0.54
MFR. E	Pass	121	103.7*	100	RRT 0.36: 0.38%* RRT 1.36: 0.24%*	0.93
MFR. F	Fail*	156*	0*	0*	NA	NA
MFR. G	Pass	121	94.4*	98.7	RRT 0.39: 0.15%* RRT 0.49: 0.12%*	0.55
Reseller A	Pass	121	94.9*	98. 6	RRT 0.49: 0.29%* RRT 1.35: 0.11%*	0.73
Reseller B	Pass	120	98.5	98.5	RRT 0.49: 0.28%*	0.73
Reseller C	Pass	121	94.5*	98.2	RRT 0.49: 0.22%* RRT 0.77: 0.13%*	0.58

* Result fails specification.

RRT, relative retention time.

Testing showed that all 11 API samples met the established acceptance criteria for appearance, residual solvents, ordinary impurities (TLC), and elemental impurities. Table 3 lists results for the tests that showed differences among the samples: identity, melting point, UV assay, unspecified impurities > 0.10%, and total impurities.

#### Identity and melting point

All samples passed the identification tests (IR and TLC) except the sample from Manufacturer F (Table 3 MFR. F), which failed both identification tests. Similarly, all samples had melting points in the acceptable range except sample F, which had a melting point of 156°C, far outside of the specification limits of 120-124°C. This provided further evidence that the sample F material, which was received in a bottle from the Chinese manufacturer labeled as HPC, was not in fact HPC.

#### UV assay

The UV assay of the control sample was 99.7%. Five of the remaining nine samples were within the 97.0-103.0% USP specification limits. Three samples were out-of-specifica-tion on the low side (i.e. <97.0%), and one sample was out-of-specification on the high side (i.e. >103.0%). The assay of sample F was essentially zero, further substantiating that the sample was not HPC.

#### HPLC assay and impurities

The HPLC assay results for all but one of the API samples were in specification, and in the range of 98.2-100.0%. The sample from Manufacturer F was again the lone exception, yielding a value of 0% by the specific HPLC assay procedure.

Two specified related substances are listed in the approved manufacturer's HPC API specifications: 17α-hydroxyprogesterone, which is a degradant of HPC, and the 177α-β-methyl-d-homo compound, which is a synthetic process impurity. All of the API samples in this investigation met the criteria for both of these specified impurities.

The approved manufacturer's specification limit for unspecified impurities in the API is NMT 0.10%, which matches the identification threshold for impurities in FDA's Guidance for Industry^26^. In the control sample, no unspecified impurities exceeded the 0.10% specification limit and total impurities were 0.19%. Excluding Sample F, which was not HPC, eight of the nine remaining HPC API samples (89%) contained one or more unspecified impurities above the approved API manufacturer's specification. Only one sample of API from China (Manufacturer C) complied with the unspecified impurities specification that the FDA-approved manufacturer of is required to meet. Table 3 lists the peaks that were above the acceptance criteria for unspecified impurities by their relative retention time (RRT) and the quantitative amount relative to the primary peak. For the API sample from Manufacturer F, the values are listed as not applicable (NA), since these impurities are expressed relative to HPC content, which for this sample was zero. Excluding Sample F, all samples met the NMT 2.0% specification limit for total impurities; however, the levels of total impurities in API samples from the Chinese sources were 2.5-4.9X higher than the level of total impurities in API from the approved source.

#### Identification of sample from Manufacturer F

Additional testing by FTIR, LC/MS and NMR was conducted to identify the sample of white powder from Manufacturer F, which the previous test results indicated was something other than HPC. The additional testing, and the original melting point, conclusively identified the unknown substance as glucose.

### HPC injection

The results for the testing of the HPC injection samples, both the control sample (FDA-approved GMP product) and the 30 compounding pharmacy samples, are listed in Table 4. The control results, for which the replicates are all the same lot number of product, are shown for both testing laboratories. These were all within expected variance from the values on the certificate of analysis for this lot, showing that the validated test methods were performing adequately upon transfer to the testing laboratories and prior to testing samples from the compounding pharmacies.

**Table 4:** Hydroxyprogesterone caproate injection - laboratory test results.

Sample source	Test laboratory	Appearance	UV assay (%)	HPLC assay (%)	Unspecified impurities > 0.20%
Control	A	Conforms	99	99.3	None
Control	A	Conforms	100.2	99.6	None
Pharmacy 1	A	Conforms	99	96.6	RRT 0.23: 0.60%*
Pharmacy 2	A	Conforms	103.9	100.6	RRT 0.23: 0.43%*
Pharmacy 3	A	Conforms	96.9	94.8	RRT 0.23: 0.26%* RRT 0.45: 0.25%*
					RRT 0.75: 0.27%*
Pharmacy 4	A	Conforms	99	96.5	None
Pharmacy 5	A	Conforms	97.7	96.5	RRT 0.75: 0.27%*
Pharmacy 6	A	Conforms	98.7	97.1	RRT 0.41: 0.23%*
Pharmacy 7	A	Conforms	97.1	95.4	RRT 0.23: 0.22%*
Pharmacy 8	A	Conforms	97.6	93.1	RRT 0.23: 0.29%* RRT 0.41: 0.31%*
Pharmacy 9	A	Conforms	101.9	99	RRT 0.23: 0.27%*
Pharmacy 10	A	Conforms	110.6*	108.6	None
Control	B	Conforms	98.6	98.5	None
Control	B	Conforms	96.4	99	None
Control	B	Conforms	93.8	99.2	None
Pharmacy 11	B	Conforms	104.9	106	RRT 0.24: 0.22%*
Pharmacy 12	B	Conforms	113.0*	109.7	None
Pharmacy 13	B	Conforms	98.6	98.1	None
Pharmacy 14	B	Conforms	129.5*	134.2*	None
Pharmacy 15	B	Conforms	98.2	99	None
Pharmacy 16	B	Fails* (visible particulates)	56.8*	55.2*	RRT 0.24: 0.72%*
Pharmacy 17	B	Conforms	98.1	97.3	RRT 0.24: 0.29%*
Pharmacy 18	B	Conforms	82.2*	88.6*	RRT 0.24: 0.21%*
Pharmacy 19	B	Conforms	100.5	100.7	RRT 1.1: 0.21%*
Pharmacy 20	B	Conforms	89.3*	87.2*	RRT 0.24: 0.34%*
Pharmacy 21	B	Conforms	252.0*	98.8	None
Pharmacy 22	B	Conforms	98.6	95	None
Pharmacy 23	B	Conforms	98.8	97.9	None
Pharmacy 24	B	Conforms	95.6	94.4	None
Pharmacy 25	B	Conforms	98.8	98.9	None
Pharmacy 26	B	Conforms	90	89.4*	RRT 0.75: 0.29%*
Pharmacy 27	B	Conforms	103.9	104. 9	None
Pharmacy 28	B	Conforms	101.1	102.1	RRT 0.23: 0.38%* RRT 0.75: 0.41%*
Pharmacy 29	B	Conforms	101.6	104.8	None
Pharmacy 30	B	Conforms	93.98	96.19	None

* Result fails specification.

RRT, relative retention time.

#### Identification and appearance

The control samples and all of the HPC injection samples from the compounding pharmacies met the identification specification. All control samples and 29 of 30 pharmacy samples met the FDA-approved appearance specification for the finished product, which requires the injection to be a clear, colorless or pale yellow solution, free of foreign particulate matter. The sample from Pharmacy 16 failed appearance due to the presence of visible solid particulates, which would also cause it to fail the general USP requirements for injections.

#### USP assay (UV)

Five replicate tests of the control sample were all within the USP assay limits of 90.0-110.0% of label claim by the UV test method. Seven of the 30 HPC injection samples obtained from compounding pharmacies (23%) were found to be outside of the specification limits when tested by this assay procedure. Three of these seven samples were sub-potent and four of the seven were super-potent, with assay results ranging from 56.8% to 252.0%.

#### HPLC assay

All five control samples were well within the approved specification for assay by HPLC (90.0-110.0% limits). Five of the 30 HPC Injection samples obtained from compounding pharmacies (17%) were found to be out-of-specification when tested by the HPLC assay procedure. Two samples (Pharmacy 10 and Pharmacy 12) that were marginally above the upper specification limit by USP assay procedure (110.6%, 113.0%) were marginally within the upper specification limit by the HPLC assay procedure (108.6%, 109.7%). One sample (Pharmacy 26), whose assay was exactly at the lower specification limit by the USP procedure (90.0%), was slightly below the lower specification limit (89.4%) when tested by the HPLC assay. For the sample from Pharmacy 21, there was a major discrepancy between the USP assay result (252.0%) and the HPLC assay result (98.8%). The USP assay result was investigated; however, no laboratory error was found, and the same result was obtained upon retesting of the sample using the official USP method.

#### Analysis of impurities

When tested according to the validated HPLC procedure, unspecified impurities in the control samples were all well below the specification limit. Sixteen of thirty samples of HPC injection made by compounding pharmacies (53%) were found to contain at least one unspecified impurity greater than 0.20%, and three of the 30 samples (10%) contained two or more unspecified impurities greater than 0.20%. Unspecified impurities exceeding this limit are listed in Table 4 for each sample, along with their RRT and their quantitative amount relative to the primary peak.

## Discussion

### Potency failures

The data show that when tested by the USP UV assay procedure, the potencies of HPC API samples from the Chinese sources do not consistently meet the required compendial standards. Except for the sample from Manufacturer F, potencies for all samples were greater than 90%, which is consistent with the positive identity test results. All of the API potency failures by the nonspecific USP method were for samples that also had out-of-specification levels of impurities. These potency failures may have been due to differing absorbances of the impurities versus the pure API, which can alter the apparent potency.

For the HPC injection samples, HPLC assay results outside of the 90-110% specification range are likely due to errors in preparation by the compounding pharmacy, since the difference is too large to be attributable to minor variations in the API potency. The percent of samples failing the potency specification and the range of the results are both consistent with the previous FDA survey of compounded drugs published in 2006^12^. Potency failures by the USP assay procedure were approximately equally distributed between sub-potent (3 failures) and super-potent (4 failures). Although there has not been a definitive dose-ranging study published for HPC for this indication, sub-potent products are of concern because of the potential for inefficacy, which in this case may cause an unnecessary and costly preterm birth. The super-potent products in this study do not approach the 1000 mg doses previously used for HPC in treatment of adenocarcinoma^27^, but the risk-benefit ratio for a drug used to treat terminal cancer and a drug given to a healthy pregnant woman are quite different. A physician prescribing a medication for reducing the risk of preterm birth certainly expects it to be the correct dose, the dose that was proven to be safe and effective by the randomized clinical trial^4^.

Numerous discrepancies were found between the UV and HPLC assay results for the HPC injection samples. Several samples from compounding pharmacies failed the required USP UV assay, but passed when tested by the HPLC procedure. This is not surprising considering the non-specific nature of the older USP procedure, which may be significantly biased upward or downward by impurities in the API. The HPC injection sample from Pharmacy 21 which had an extremely high assay result (252.0% of label claim) was retested by the USP UV assay, and the result was verified. However, the more specific HPLC assay of this sample was on-target (98.8%). The high assay result was possibly due to differences in the inactive ingredients used in this preparation, which interfered with the USP procedure. Compounding pharmacies often modify a formulation for specific patients, based on individual needs, although there are no assurances that such changes do not alter the absorption or stability of the formulation, and there was no indication why the formulation may have been altered in this case.

### API identification failure

The fact that a purported source of HPC for human use could be imported to the United States and found to instead be glucose is a serious concern. It is unknown whether this was due to a label mix-up in a factory not following GMP procedures, a counterfeit sample, or purposeful substitution for profit (i.e. economically motivated adulteration). This is not the first instance of adulterated materials originating from unregulated Chinese sources, as recent events involving heparin and melamine have been reported^28^,^29^. Drug importers and resellers in the United States often contribute to the difficulties of tracing an API to its original synthetic source by repackaging and relabeling the material under their own name, and removing information provided by the original manufacturer. Thus, compounding pharmacists may in good faith believe they are using API from a regulated US source, when in fact the material was originally produced in an unregulated Chinese facility. In the absence of FDA oversight, the professional liability falls on the pharmacist and prescribing physician to verify the source and quality of the API used to compound the drug product. We were unable to verify any authentic US sources that were original manufacturers of HPC API; all samples except the control originated from unregistered Chinese sources.

### Impurity failures

There were unspecified impurities above the FDA-approved specification limits in nearly all of the API samples from the Chinese sources, and only one of the ten API samples would have been acceptable for use in the FDA-approved drug product. The structures, and therefore the resultant toxicities, of the unspecified impurities in the API samples are unknown. These impurities may arise from the API synthesis, which may follow a different synthetic route than the FDA-approved API and therefore have a different impurity profile. In the absence of validated cleaning procedures, cross-contamination of the API from other chemicals synthesized in the same equipment cannot be ruled out.

The majority of the HPC injections from compounding pharmacies had impurity levels higher than those found in the control product. These could arise from API impurities carried over into the compounded injection, or from residual contaminants left over from inadequate cleaning of equipment after compounding other prescriptions. In addition, the method used by compounding pharmacies to sterilize HPC injections may contribute impurities to the drug product in the form of degradants. The FDA-approved product is made using aseptic techniques and sterile filtration at controlled room temperature, whereas many compounding pharmacies use terminal sterilization procedures utilizing heat, such as autoclaving. Heat treatment of the control product for 150 min in a 150°C convection oven, which is typical of terminal sterilization procedures used in compounding pharmacies, produced a 55% increase in the area of the impurity peak at RRT 0.26, and the introduction of two additional impurity peaks at RRT 0.38 and RRT 0.52 which were not present in unheated samples. In the case where a compounding pharmacy uses a process generating impurities that are not present in the FDA-approved product, these impurities have not been characterized for safe use levels.

In an FDA-regulated product, impurities must be identified and qualified before safe specification limits can be established. Since impurities have no therapeutic value, and can potentially be harmful to a patient, considerable discussion by various glob al regulatory authorities went into setting the acceptable use limits for unknown impurities in the FDA and international guidances. Unfortunately, no such requirements exist for pharmacy compounded products. The outdated TLC impurity test in the current USP monograph for HPC API is inadequate to detect most impurities, and there is currently no impurity test at all in the HPC injection monograph. A developing fetus is more susceptible to drug-related toxicities than an adult^30^; therefore it is especially important to ensure acceptable purity of the drugs used in pregnant patients.

### Limitations of the results

The results obtained in this investigation are a snapshot obtained from certain API manufacturers and compounding pharmacies at a particular time. Subsequent investigations could yield different results, either better or worse, but these results are consistent with previous similar investigations that show significant variability in the product from compounding pharmacies. The identity of the unspecified impurities (and hence their toxicity) could not be established given the small amount of sample available in this investigation from each pharmacy. Sample size limitation also precluded investigation of the microbial integrity of the compounded products. Given the number of issues identified with potency and purity, and previous literature findings in this area^9^–^22^, it cannot be presumed that these samples would all meet the required microbial and endotoxin specification.

## Conclusions

The results that were obtained in this study demonstrate variability in the quality of HPC injection prepared by compounding pharmacies, as well as variability in the quality of the HPC API that is available to make the compounded preparations. Some of the compounded HPC injection preparations are of sub-standard quality and could potentially be considered adulterated under the Federal Food, Drug and Cosmetic Act section 501(b). These results reaffirm the role FDA plays in ensuring the quality of drugs sold in the US, and why many healthcare professionals do not recommend compounding in situations where an FDA-approved drug is available^10^,^31^–^32^.

This variability in quality is essentially invisible to patients and health care practitioners, who generally assume that all of the drugs they receive from the pharmacy conform to the same high standards of quality, and may incorrectly believe that compounded drugs are the same as generic drugs. Generic drugs are FDA-approved, and are manufactured under GMP regulations. Compounded drugs are not FDA-approved, and compounding pharmacies are exempt from GMP regulations. Not all compounded products have quality problems, and not all FDA-approved GMP-manufactured products are free of quality defects, but history has shown that GMPs significantly reduce the probability of errors in FDA-approved medications.

ACOG and SMFM have recommended that physicians be aware of the inherent differences between the FDA-approved product and compounded 17P^33^. The availability of an FDA-approved product, with consistent potency and purity, significantly alters the previous risk-benefit ratio in favor of using the approved drug. In reference to Makena, FDA has reiterated its opinion that approved drugs generally offer a greater assurance of safety compared to compounded versions^34^. There may be a misperception among some physicians that using a pharmaceutical preparation prepared by a compounding pharmacy is similar to using an FDA-approved drug for an off-label indication. However, these practices cannot be equated. Physicians should recognize that using an unapproved drug that is not made in accordance with FDA quality standards may carry additional risks, and consider whether proper informed consent should include disclosure to the patient of potential quality differences between the FDA-approved product and the compounded versions of HPC Injection.
